# Effect of 12-Week Functional Training Intervention on the Speed of Young Footballers

**DOI:** 10.3390/ijerph17010160

**Published:** 2019-12-24

**Authors:** Jakub Baron, Anna Bieniec, Andrzej S. Swinarew, Tomasz Gabryś, Arkadiusz Stanula

**Affiliations:** 1 Institute of Sport Science, The Jerzy Kukuczka Academy of Physical Education, 40 065 Katowice, Poland; jakubbaron95@gmail.com (J.B.); andrzej.swinarew@us.edu.pl (A.S.S.); 2 Department of Tourism and Pro-Health Physical Activity, The Jerzy Kukuczka Academy of Physical Education, 40 065 Katowice, Poland; a.bieniec@awf.katowice.pl; 3 Institute of Materials Science, Faculty of Science and Technology, University of Silesia, 41 500 Chorzów, Poland; 4 Department of Physical Culture, Faculty of Health Sciences, Jan Długosz University, 42 200 Częstochowa, Poland; tomaszek1960@o2.pl

**Keywords:** functional training, functional movement system, movement patterns, speed, football

## Abstract

The aim of the research was to verify the functional state of young football players using selected tests of the Functional Movement Screen (FMS) protocol, as well as the impact of the 12 weeks of functional training on the speed parameters. The research was conducted on 20 highly competitive young (U17) football players. Research project was conducted in two stages: in the first part of the study, the functional assessment was made by using the FMS test, then the measurement of the speed parameters was done with the Microgate photocells system. Results showed a significant improvement in the functional state of young football players: FMS 1 (45.2% of difference, *p* = 0.004), FMS 2 (24.3% of difference, *p* = 0.012), FMS 3 (48.5% of difference, *p* = 0.001). After the functional training program, there was also an improvement in the parameters of the acceleration and velocity: acceleration between 5–10 m and speed between 10–30 m shows significant improvement (expressed during covering a given distance) of the footballers, amounting to 0.02 s (2.4%) and 0.04 s (1.5%). But there was no improvement in acceleration between 0–5 m. An appropriate training schedule, based on FMS results, should be adopted in the annual training program to improve basic motor skills of the football players and minimize their injuries.

## 1. Introduction

Football is a team sport that demands endurance and speed and involves short sprints, rapid accelerations, decelerations, quick changes in direction, jumping and tackling and many other unorthodox movements [1,2]. The ability of players to create actions at a very fast pace has a significant impact on the course and result of the game. Actions that require maximum speed, acceleration or agility are considered to be very fast-paced. At the same time, it was noticed that maximum efforts account for only 11% of the total game time. Thus, the proper use of motor skills is crucial in the context of losing or winning a match [3]. On average, football players cover a distance of 9–12 km during the match, of which they spend only a little time on maximum efforts. Football sprints during the game oscillate around 17–29 times depending on the pace of the game, tactics, opponent or position on the pitch. Speed can be defined as the ability to overcome the distance in the shortest time, accelerating from one point to another in high velocity [4]. Competitors usually perform sprints lasting 2 to 4 seconds over a distance of 20-m or less [5]. Carling et al. [6] noted that the average sprinting distance in a football match is 16.5 meters. Sprints in football are most common in goal situations for both the possible scorer and the supporting player(s) [7,8].

Starting speed, acceleration and maximum speed are key elements in football performance [9]. Haugen et al. [10] indicated that the influence of speed performance parameters is very important in football. While performing start speed and acceleration phases athletes benefit from triple extension position further; uses propulsive force of plantar flexors, knee and hip muscles [9], which may be related to support optimal movement patterns. Research conducted by Williams et al. [11] demonstrates that changes in football speed efficiently occur as a result of increased muscle mass, changes in muscle-tendon architecture and by improving neural control and coordination. Rabita et al. [12] noted that the capability of an athlete to generate maximal power during sprint acceleration depends mainly on his neuromuscular and mechanical properties of the musculoskeletal system and his technical sprint ability to move body mass forward. It is important to know, that speed is connected with genes, related to muscle fibers and the nervous system. Speed training should be develop in training very early in young athletes, when the nervous system is adaptable 7–9 years, and also 13–16 years [13].

Football is a body-contact game that involves a high risk of injury. Research conducted among young football players showed that almost 40% of football players playing at various game levels had non-mechanical injuries in lower limbs [14]. Most of them involve hamstrings strains, traumas of the tendons and ligaments of the knees [15,16,17,18]. Moreover Ekstrand et al. [19] conducted research among 23 football teams in which they stated that, the possibility of injury is around 8 per 1000 hours of play, especially in competitive matches. A popular research tool for functional status assessment is Functional Movement System (FMS) which was created to evaluate the risk of injuries among athletes through the analysis and scoring of movement patterns especially in football [20]. FMS is being more attractive to scientists. The researches associated with FMS protocol involving recreational, college and Olympic athletes are increasing [21]. Functional training should be fundamental to developing special and tactical skills. The essence of functional training is an individual approach and the form of training should be as close as possible to conditions that the player encounters during training or in a match. The key role in functional training is to achieve and maintain an optimal balance between mobility and stability while performing fundamental movement patterns with accuracy and efficiency [21,22]. Campa et al. [23] suggested that a good correlation between strength of the muscles, stability, flexibility and motor control is decisive to achieve and support optimal sports performance. High body mass index (BMI) and high body fat are detrimental to efficient movement patterns [24].

The aim of this study is to verify the functional status of young footballers using selected components of the FMS protocol, as well as to assess the impact of 12 weeks of functional training on selected speed parameters (acceleration, maximum running speed) of young footballers. The novelty of this investigation was to determine if improving functional assessment could improve special fitness ability (acceleration and full speed), which are key elements in football.

## 2. Materials and Methods 

### 2.1. Research Design

The research was carried in two parts: the first during the time of preparation for the autumn season and the second just after the autumn round of matches, directly before the preparation for the spring round. No further activities were carried out before test day, to guarantee the athletes an optimal recovery. The research was conducted in two stages: In the first stage, the functional assessment and anthropometric measurements were recorded. All the speed parameters were measured in the second stage. All tests were performed from between 8.00 a.m. and 12.00 p.m., keeping the order of approaching competitors to the test trials unchanged. All the tests were conducted in the sports hall in Chorzów (Poland).

### 2.2. Subjects

All the participants (n = 20) of this study were young football players of below 17 years of age (U17). They were the inmates of the Silesian Football Academy and participants of the Central Youth Junior League (Table 1).

Before participating in the research, each participant was informed about the purpose of the research and confidentiality of results. The participants were given the freedom to withdraw their participation from the study without any consequences. The inclusion criterion was: consent for participation in the research, good health and active participation in functional training. The exclusion criteria were: lack of consent of the trainer, injury or illness and rehabilitation not allowing the participant to participate in regular training. Written informed consent was signed by the parents of minors before participation. The study was approved by the Ethics Committee of The Jerzy Kukuczka Academy of Physical Education in Katowice (No. 8/201).

### 2.3. Anthropometric Measures

All of the anthropometric measurements were taken according to standard methods. Height (to the nearest 0.1 cm; Harpenden Portable Stadiometer, Holtain, UK), body mass (0.1 kg) and percent body fat (0.1%) with a total body composition analyser (TANITA BC-420S MA, Tokyo, Japan). The participants wore light indoor clothing and were barefoot when they were measured. The same investigator made all anthropometric measurements. Body mass index (BMI) was calculated as the ratio of body weight to height squared (in 1 kilogram per square meter). Data for each component are presented in Table 1.

### 2.4. Functional Screen

The FMS test is a comprehensive screening tool that assesses the quality of basic movement patterns and identifies movement limitations and asymmetries [25]. The FMS test is popular in the coaching environment, mainly due to its simplicity and easy to use equipment. Fundamental movement patterns were tested which are the basis for more efficient sports movements [26]. In the conducted studies, global movement patterns were assessed, which require appropriate mobility, stability and range of motion in the joints as well as balance. Three tests were used to assess global movement patterns: 1) deep squat—which is a movement needed in most athletic events and is required for most power movements involving the lower extremities. The deep squat is a test that challenges total body mechanics when performed properly. The deep squat is used to assess bilateral, symmetrical, functional mobility of the hips, knees, and ankles. The dowel held overhead assesses bilateral, symmetrical mobility of the shoulders as well as the thoracic spine. 2) hurdle step—designed to challenge the body’s proper stride mechanics during a stepping motion. The movement requires proper coordination and stability between the hips and torso during the stepping motion as well as single leg stance stability. The hurdle step assesses bilateral functional mobility and stability of the hips, knees, and ankles. 3) in-line lunge—attempts to measure body position during rotational, decelerating, and lateral type movements. The in-line lunge is a test that places the lower extremities in a scissor style position challenging the body’s trunk and extremities to resist rotation and maintain proper alignment. This test assesses hip and ankle mobility and stability, quadriceps flexibility, and knee stability. Each test is evaluated on a four-point scale from 0 to 3, where 0 means pain during the test, 1—inability to perform the test, 2—perform motion with compensation, 3—correct movement [25]. Individual tests were carried out three times and evaluated for the best test. In case of doubt, the rating was downgraded. The researchers evaluated the movement in the frontal and sagittal plane and score was assigned independently for left and right sides of the body for in-line lunge test and hurdle step test. The overall score was the lowest score from the 2 sides. The FMS evaluation is done without warm-up and athletes wear sporty outfits and footwear. Specialized equipment “FMS Test KiTM” was used to perform the test. The kit includes a base—a board with dimensions of 5 × 15 × 150 cm, tubes with a centimeter scale and rubber cord [25]. In these studies, the assessment of global movement patterns was carried out by a physiotherapist with FMS certification and by a strength & conditioning coach.

### 2.5. Speed Performance Tests

The speed test was carried out at the sports hall. The assessment of the maximum speed and acceleration was made during the run from the own command at a distance of 5 and 10 m and during a flying start at a distance of 20 m after earlier acceleration at a distance of 10 m. Measurements were made using a set of RaceTime 2 photocells made by Microgate (Bolzano, Italy ) on the playing field in a straight line, creating measuring gates on 5, 10 and 30 meters. Before testing standardized warm-up including jogging, dynamic stretching, muscle activation and mobilization of joints has been done and also speed exercises and athletic drills were carried out. After warm-up, participants had a 5 min break. Then athletes had two trials, of which the best was recorded. Sufficient recovery time of 3 min was allowed between each performance trial.

### 2.6. Functional Training Program

The purpose of the intervention was to focus on functional deficits that can occur in three FMS trials. In the first test—deep squat: the purpose was to improve squat pattern by increase the mobility of the ankle, mobility of the thoracic spine, shoulder girdle and also to improve hip function and body control. Second test—hurdle step: the aim was to increase hip mobility and improve central stabilization. In the third test—in-line lunge: the purpose was to achieve greater mobility in the ankle, knee and hip joints as well as postural control. The implemented program was planned in accordance with the FMS concept and based on functional training implemented of selected sports disciplines [20,27] as well as on the experience of coaches and physiotherapists (Table 2). Each session was supervised by a certified strength and conditioning coach.

### 2.7. Statistical Analysis 

Descriptive statistics (mean and standard deviation) were calculated. Before using parametric tests, the assumption of normality and homoscedasticity were verified using the Shapiro–Wilks W-Test. In order to compare the data from the acceleration and running speed obtained before and after the implementation of the training program, a paired sample t-test was used. Due to the step nature of the FMS test results, the Wilcoxon signed-rank test was used to evaluate the comparison of results recorded before and after the experiment. For all analyzes, a level of *p* ≤ 0.05 was selected to indicate statistical significance. All calculations were done with the STATISTICA –statistical package ver. 13.3 PL of TIBCO Software Incorporation (Palo Alto, CA, USA).

## 3. Results

### 3.1. Functional Status of Global Patterns in the FMS Test

The assessment of the functional status of young players conducted before the training program indicates (Table 3) that in the FMS 1 test the average score was 1.6 ± 0.51 (scale from 1 to 3). The calculated skewness coefficient of −0.21 indicates that the majority of respondents achieved a score higher than the average. In the case of the FMS2 test, the average value for the whole group was 1.9 ± 0.49 and, similarly to the FMS1 test, the majority of people achieved a score higher than the average for the whole group (AS = −0.442). The average result for the whole group for the FMS3 test was 1.7 ± 0.59. It should be emphasized that in test 3, the results of the examined persons were the most diversified, which was confirmed by the calculated coefficient of variation (V = 35.6%). Contrary to the FMS1 and FMS2 test, the majority of respondents obtained a score lower than the average calculated for the whole group.

### 3.2. Influence of the 12-Week Functional Training Program on the Functional State of Global Patterns in the FMS 

The 12-week functional program was aimed to improve selected motor skills among young footballers. It is interesting to what extent the change occurred in global patterns in the FMS test. Based on the results presented in Table 4, it can be concluded that in the FMS1 test there was a statistically significant (*p* = 0.004) improvement in results by 45.2% (1.55 vs. 2.25). To a slightly lesser extent, but also statistically significant (*p* = 0.012) there was an improvement of 24.3% in the FMS2 test (1.85 vs. 2.3). The highest progression was recorded in the FMS3 test, where the difference between the measurement before and at the end of the training program was 48.5% (1.65 vs. 2.45, *p* = 0.001).

### 3.3. The Impact of the 12-Week Functional Training Program on Improving Running Speed and Acceleration

The applied 12-week functional training did not improve the acceleration of the football players in the range of 0 to 5 m (Table 5). Before the introduction of training, the competitors covered the distance of the first 5 m in 0.96 ± 0.06 s, while after completing the functional training program their time deteriorated by 0.02 s (1.7%). The measurement of the speed from a flying start between 5 and 10 m and between 10 and 30 m allows to state that there was a statistically significant improvement in the maximum speed (expressed during covering a given distance) of the tested competitors, amounting to 0.02 s (2.4%) and 0.04 s (1.5%). On the other hand, over a distance of 30 m, the tested players achieved a slight improvement in time of 0.04 s (0.9%). The results of the 30 m run calculated in relation to the speed achieved before joining the 12-week training program and after the implementation of this training amounted to 26 km/h and 26.24 km/h respectively, which indicates a speed increase of 0.24 km/h (0.09%). Detailed results for the running tests are presented in Table 5.

## 4. Discussion

The main purpose of this study was to verify functional status and speed parameters and then to introduce the intervention functional training program, which may improve the functional status and speed parameters. As hypothesized, the functional status after training intervention have improved significantly. Moreover, acceleration (5–10 m) and top speed were improved significantly. There was no significant difference in acceleration 0–5 m after training intervention. 

Training overload is common in youth sports and it is likely to raise the number of injuries. Neglect in the first stages of functional development of youth athletes may result in the lack of adequate axiality of the body segments, leading to compensation, excessive overloads, improper transmission of force, for example in the case of a sudden change of direction, acceleration, braking. Such excessive exploitation of athletes speeds up the risk of injury and reduces its motor potential. Deficiencies in functional fitness is more susceptible to loads and likely to cause more injuries [21,22]. Adult athletes have problems with muscular dystonia, contractures, posture defects, and degenerative changes in the joints. These can be irreversible changes that an athlete can struggle with even after his career [21,22]. This is especially important because of the greatest physical development between junior (U17) and adolescent age (U20) [28]. The FMS test was created to assess the risk of injury for football players, and as a screening tool to assess basic movement patterns and possibly occurring movement compensations and asymmetries. Deficiencies in basic motor skills (coordination, mobility and stability) can translate into limitations in directed motor skills (strength, speed, power, endurance) as well as further on special fitness in a given discipline [25,27]. Cook [29] emphasized that a player with functional movements may show better skills and techniques of movement. In contact sports such as football, reactive agility or speed of reaction are favorable skills for players because they translate into the possibility of using pressing on the rival, getting or keeping possession of the ball [30]. Newman [31] also has a similar opinion and believes that football players, as well as other team game players, perform many sprints and accelerations during the match. Speed and acceleration are also very important factors in sports played on pitch, due to the maximum speed of short distances, which according to Baker [32] and Sayers [33], is the key to success. Gabryś et al. [34] and Stanula et al. [35] stated that assessment of maximal running speed is a more important factor of athlete’s performance than VO_2_max and other physiological variables. The importance of functional possibilities in developing special efficiency can be illustrated by research, which showed that proper tilt of the torso, foot placement, regulation of the running step and running posture are important technical factors regarding reactive agility, which are extremely important in one-on-one situations [36].

The first FMS test—squat—evaluates seven functional components [37]. The first assessment of the test showed lots of deficiencies in the proper performance of testing movement patterns. Mobility of the ankle, hips and shoulder girdle were assessed as the worst dysfunctions detected during the FMS test. The functional training program was aimed to correct dysfunctions and was supposed to improve the quality of movement patterns. Based on the results presented in Table 3, it can be stated that the results have improved in all tests. The highest progression was recorded in the FMS 3 test, where the difference between the measurement after the training program and before was 48.5%. 

Since the results obtained in acceleration phase tests between 5–10m and 10–30m, velocity and overall time at the distance, have improved, it can be concluded that the improvement of the functional state causes the progression of running speed. Based on tests carried out after the training program in comparison with the initial tests, it was found that the early phase of acceleration did not improve. The essence of using functional training based on movement patterns is confirmed by the research of Campa et al. [38] who believed that better movement patterns may improve speed performance. Attention should be paid to the fact that wrong movement patterns negatively affect the ability to perform fundamental movement patterns with precision and appropriate efficiency. They also increase the risk of injury [23]. High percentage of asymmetrical and dysfunctional movements can be corrected with specific interventions [20,23,39]. 

There are scientific studies that shows the correlation and importance of introducing functional assessment and functional training program. Hong-Sun Song et al. [40] tested baseball players for functional assessment by FMS test, in which they measured functional status, strength and flexibility also. A significant impact of the training program on the improvement of functional assessment has been demonstrated, which is associated with a lower risk of injury and the probability of increased strength in the subjects has been demonstrated, and it is possible to increase flexibility, although no significant correlations were found. Similar opinion about positive aspects of functional training program have Stanek et al. [41] and Kiesel et al. [42] who stated that, individualized corrective exercise program was effective at improving scores on the FMS and minimize injury risk.

In the other hand, there are few researches, that shows no correlation between intervention program and FMS. For example, Dossa et al. [43] suggest that, FMS cannot be recommended as a pre-season screening tool for injury prevention in population of junior hockey players. Also the study by Venter et al. [44] concluded that FMS score is not a good indicator of the physical readiness or performance of female netball players.

We suggest that, functional training aimed at shaping fundamental motor skills or eliminating functional limitations (pre-hab) can be a good tool to supplement training at various stages of the macrocycle. The presented results should lead to considerations to apply a longer training program.

The practical implication from this study and the novel finding that could help coaches, strength & conditioning coaches to improve their training systems is that the use of FMS test trials can help reveal inferior movement patterns and then improving quality of global movement patterns. Introducing functional training program in regular microcycle may protect the player from injury and improve their speed parameters.

## 5. Conclusions

The aim of this study was to verify the functional state of global movement patterns and the level of running speed and acceleration of young footballers after the introduction of the 12-week training intervention. The training program was aimed at improving the condition of selected movement patterns. It should be emphasized that the functional condition of the examined players has significantly improved. Running speed after training intervention has also improved although no such improvement was noted in the acceleration. Attention should be paid to the essence of implementation of training intervention and monitoring of players in terms of correlation of functional state with the level of running speed and acceleration and other motor skills.

The purpose of subsequent studies should be to introduce an intervention program and compare it with the control group. Another purpose of research should be the implementation of an intervention program based on the ideology of FMS and the use of elements of special efficiency; especially based on acceleration and speed.

## Figures and Tables

**Table 1:** Characteristics of basic somatic parameters of the tested group of players (n = 20)

Parameters	m ± SD	Me	Range (min–max)	V	As	Ku
Age	16.8 ± 0.6	17	15–17	3.4	−3.14	8.61
Height (cm)	175.7 ± 6.4	176	167–187	3.7	0.21	−0.87
Body weight (kg)	66.5 ± 7.4	64	54.8–81	11.1	0.48	−0.85
BMI (kg/m^2^)	21.5 ± 1.8	21.62	18.8–24.6	8.5	0.20	−1.16
FAT (%)	12.6 ± 2.2	12	9.5–15.5	12.9	0.31	−0.09

m ± SD: mean and standard deviation; Me: median; Range: the smallest and largest value; V: coefficient of variation; As: skewness coefficient (asymmetry); Ku: coefficient of focus (kurtosis).

**Table 2:** A detailed description of the training program implemented in the research group.

Part of the Training Unit	Exercises/Goal/Execution (Volume)
Warm-up (15–20 min)	Running (4’) Run with the delivery and delivery step (2 × 20 m) (30’’) Cross step (interlace) (2 × 20 m) (30”) Forward shoulder circulation (30’’) Back arm circulation (30’’) Dynamic stretching (5’) Hip rotation (2 × 10 rep) (1’) twist with a trunk torsion (2 × 5 rep) (1’) Buttocks Activation with a mini band (2 × 10 rep) (2’) Skip A (10 m) (10’’) Skip C (10 m) (10’’) Running rhythm (15 m) (10’’)
Main Part (45–60 min)	Back Stretching exercises (2–3 ex.) Mobilization of shoulder complex (2 ex.) Mobility of the thoracic spine.: Extension of the thoracic spine (1–2 ex.) Four - point kneeling position. Thoracic spine rotation (2 ex.) Hip mobilization in the direction of flexion, extension, external and internal rotation (5 ex.) Ankle Mobilization towards the dorsiflexion (2 ex) Central stabilization, stabilization of the ilio-lumbar-pelvic complex (3 ex.) Exercises with bands, global patterns (2 ex.) Balance and coordination exercises (2 ex.)
Cool Down (10 min)	Foam rolling (10’)

**Table 3:** Characteristics of the results of the functional status of movement patterns carried out prior to the start of the training.

Variables	m ± s	min–max	±95% PU	V	As	Ku
FMS 1	1.6 ± 0.51	1–2	1.3–1.8	32.9	−0.218	−2.183
FMS 2	1.9 ± 0.49	1–3	1.6–2.1	26.5	−0.442	1.304
FMS 3	1.7 ± 0.59	1–3	1.4–1.9	35.6	0.212	−0.552

**Table 4:** Results of the functional status assessment of the functional status of the tested group of players (n = 20). The significance of differences was assessed using the Wilcoxon test.

Variables	Pretest	Posttest	Difference (%)	*p*-Value
FMS 1	1.55 ± 0.51	2.25 ± 0.55	−0.70 (−45.2%)	0.004
FMS 2	1.85 ± 0.49	2.30 ± 0.57	−0.45 (−24.3%)	0.012
FMS 3	1.65 ± 0.59	2.45 ± 0.69	−0.80 (−48.5%)	0.001

**Table 5:** Results of the running speed and acceleration tests in the group of football players (n =20)**.**

Variables	Pretest	Posttest	Difference (%)	*p*-Value
0–5 m (s)	0.96 ± 0.06	0.97 ± 0.06	−0.02 (−1.7%)	−1.001 (0.329)
5–10 m (s)	0.73 ± 0.04	0.72 ± 0.04	0.02 (2.4%)	2.438 (0.025)
10–30 m (s)	2.47 ± 0.1	2.44 ± 0.1	0.04 (1.5%)	4.188 (0.001)
0–30 m (s)	4.16 ± 0.17	4.12 ± 0.17	0.04 (0.9%)	1.832 (0.083)
Velocity (km/h)	26.00 ± 1.04	26.24 ± 1.12	−0.24 (−0.9%)	−1.863 (0.078)
